# Editorial: Home Cage-Based Phenotyping in Rodents: Innovation, Standardization, Reproducibility and Translational Improvement

**DOI:** 10.3389/fnins.2022.894193

**Published:** 2022-06-23

**Authors:** Stefano Gaburro, York Winter, Maarten Loos, Jeansok J. Kim, Oliver Stiedl

**Affiliations:** ^1^Tecniplast S.p.A, Buguggiate, Italy; ^2^Institute for Biology, Humboldt University, Berlin, Germany; ^3^Sylics BV, Utrecht, Netherlands; ^4^Department of Psychology, University of Washington, Seattle, WA, United States; ^5^Department of Health and Environment and Center for Neurogenomics and Cognitive Research, Vrije Universiteit (VU) Amsterdam, Amsterdam, Netherlands

**Keywords:** home cage monitoring, cognitive function, locomotor activity, animal welfare, human disease models, early disease symptom detection

Novel and emerging technologies, such as home cage monitoring (HCM) systems, permit 24/7 collection of behavioral data under undisturbed conditions. HCM minimizes the impact of stressors that arises from human interaction or the testing in novel test environments, which may bias readout parameters (science) and/or affect animal welfare since science and welfare are highly interdependent. There is increasing evidence that laboratory animals living under enriched and group-housed conditions display a behavioral repertoire that is much richer as compared to what is observed in classical behavioral experiments (e.g., open field, total distance traveled and number of center/corner visits). Behavioral phenotyping experiments performed by classical tests show limited replicability due to idiosyncratic results to a particular laboratory (Crabbe et al., 1999). With this editorial about our Research Topic we try to highlight advances in technologies in HCM that are relevant for animal welfare, scientific aspects or both, such as the development of novel biomarkers (see Baran et al.) that can lead to better translational approaches in different animal welfare and scientific areas as described below.

## Definition of HCM Systems

HCM occurs in a cage where the animal spends more than a limited period of time (≥24 h) to either perform certain behavioral tasks or display physiological responses. However, proper HCM should be differentiated from benchtop technologies as highlighted by Baran et al. The need for a consensus across the neuroscience community recently led to the initiation of two work groups mainly in North America (https://www.na3rsc.org/tdb/a) and Europe (https://www.cost.eu/actions/CA20135/) that now try to harmonize the definitions of HCM, provide examples, generate guidelines and try to identify standards for improved comparability of results and new developments.

## Reproducibility and Methods in Behavioral Science

The reproducibility crisis in animal research is a well-known issue (Richter and von Kortzfleisch, 2020). Can automatization increase reproducibility of animal research? Richter suggests that controlled heterogenization in combination with HCM may lead to increase constructive and predictive validity. Along the line of data reproducibility, Engelbeen et al. tried to replicate findings in the mdx mouse model of Duchenne muscular dystrophy by testing mice in a standard battery of behavioral tests using both conventional and HCM methods. In contrast to previous findings using only female mdx mice, a learning deficit could not be reproduced suggesting an interaction of genetic and environmental factors contributing to cognitive performance differences in mdx mice.

According to Voikar and Gaburro and Baran et al., a combination of different technologies can help identify biomarkers that have the potential for transfer to the clinic. Including data that cover 99% of an animal's time and obtaining such data from the more complex situation of group-housing may increase the power of such approaches. New challenges range from 24/7 data management, data analysis tools (software tools), interpretation of large, unstructured data sets up to IT/cybersecurity issues. This means that not only scientists are necessary in a successful implementation of HCM systems.

Behavioral video monitoring has a long history as shown by Baran et al. and in the review by Grieco et al. These articles provide an overview of the use of video monitoring, the interpretation of data generated, and the scope that can be obtained from an expanded use of video monitoring technology. More recent developments advancing this technique are explained by Gharagozloo et al. who show that complex and more naturalistic behaviors can be studied through machine learning approaches and artificial intelligence.

Alternatives to video monitoring with specific applications to learning and memory are described by Voikar and Gaburro, other technologies are applied in substance use disorders as described by Iman et al. Specifically, RFID-based technology to recognize event-based animal behavior can be employed. Such technology allows interpretations in the context of reward learning and more complex behaviors such as sociability. The use of RFID transponders in group-housed mice also allows to study lifetime changes in animal behaviors in normal and pathological conditions which may address important scientific questions such as the interaction between cognition and aging Kahnau et al. which is fundamental especially in neurodegenerative disease progression.

### Animal Welfare

Four articles address the connection between animal welfare and behavioral experimental outcome. This includes using behavioral outcome to classify the severity level of a treatment in the context of animal welfare. In fact, simple routine operation such as cage-change can increase physiological parameters (heart rate, locomotion) for 90 min up to a few hours (Stiedl et al., 2004; Pernold et al., 2019). Analyzing the physiological response, specifically heart rate, to evaluate the impact of surgery with radio-telemetry for a neuroscience rat model, was important to assess when the animals could be exposed to a battery of behavioral tests as reported by Wassermann et al. A follow-up study from the same group by Zentrich et al. used spontaneous activity as marker of animal welfare. Here, an acute experimental colitis mouse model, starting from the highest level of severity as established by a clinical score (10 different parameters), expressed a reduction in locomotion that remained significantly suppressed until the mice recovered from the treatment indicating that HCM can be used for routine severity assessment in biomedical research.

The effect of ambient illumination on the behavior of animals has not been systematically studied in the animal facility where animals are bred and kept. Yet, it is well known that light conditions can dramatically affect the expression of various behaviors in visual behavior tests. Therefore, Steel et al. systematically evaluated the spontaneous activity depending on the position of the cage in the rack. Cage position relative to the position of the room lights had a dramatic effect on 24/7 activity in the HCM as measured by different parameters. Single parameters differed up to 15-fold between cages in top (higher light intensity) vs. bottom rack position (lower light intensity). For cage position from left to right there was still a 3-fold difference in light intensity. This suggests that for increased uniformity and reproducibility of cage rack studies, each cage should have its own controllable light source. Interestingly, animals in red tinted cages, which block most visible light, did not display such cage variation in activity between cage locations. However, they showed a reduced diurnal activity level as compared to animals in standard transparent cages. This shows trade-offs between positive aspects for animals vs. care takers and visual daily checks.

Moore and Brook used HCM-derived activity to assess the effect of replacing an individual companion female by a different one on spontaneous locomotion of males in their cage. Using such technology revealed that replacing a female resulted in an increase in activity for up to 4 h. This was followed by a compensatory reduction in activity during the subsequent dark phase as potential sign of fatigue. A resulting refinement suggestion is that cage mate replacements should occur toward the end of the light-phase when animal activity increases, probably impacting less on the normal circadian activity.

### Neurodegenerative Models

Despite differences in their clinical manifestations, neurodegenerative diseases such as Alzheimer, Parkinson and others share common symptoms both at the clinical and at the preclinical level. An example are alterations in resting bouts in a model of Amyotrophic lateral sclerosis (ALS). Here, resting bouts were used as an index of reduced sleep to establish a novel biomarker called rest disruption index (RDI). The RDI indicates the reduction of sleep in SOD1G93A mice at 16–18 weeks of age. This finding correlates with symptoms in typical behavioral tests such as the grid test as described by Golini et al.

Another approach based on a HCM with attached automated figure-8-maze (F8M) was used to test transgenic APPswe/PSEN1dE9 (APP/PS1) mice in the delayed alternation task to gain water as reward in a longitudinal manner from 2–6 months of age by van Heusden et al. Only 6-month-old transgenic APP/PS1 mice displayed an increase in the number of consecutive incorrect responses. The F8M approach indicates the possibility to monitor performance repeatedly over months. However, the expected cognitive decline was not detected as early as expected, since an earlier study (Vegh et al., 2014) reported the onset of cognitive impairment in the water maze task at 4 months of age in APP/PS1 mice. Particularly in animal models with increased anxiety-like phenotype such as APP/PS1 mice, deficits in tasks such as the water maze (Wolfer et al., 1998) may be the result of confounding effects of emotionally challenging experimental conditions on cognition in complex tasks (Diamond et al., 2007). Thus, HCM-based approaches are better suited to minimize unspecific effects of emotionally challenging conditions on cognition as demonstrated by another elegant HCM approach in 3-month-old APP/PS1 mice with higher sensitivity of visual discrimination to prefrontal cortex dysfunction than water maze performance (van den Broeck et al., 2021).

### Learning and Memory: Visual, Olfactory and Auditory Cues

Visual and olfactory cues are typically very difficult to be analyzed within HMC systems. In one of the very first articles of our Research Topic Wooden et al. the researchers substantially improved the object recognition test by applying an inexpensive 3-step approach that allows to adjust the experimental conditions according to the model for providing more robust results.

Advanced testing procedures in the learning, memory, and cognitive domain are based on single animal performance in an operant chamber. When an operant chamber is connected to the home cage, a form of behavioral enrichment is achieved that simultaneously allows 24/7 voluntary testing of RFID-tagged mice. This approach of Caglayan et al. used olfactory stimuli to investigate learning set acquisition. Full automation in this context eliminated human interaction and permitted individualized training schedules where a specific mouse could enter to the next level of training based on individual performance. This successfully allowed investigating higher-order cognitive function in the home cage.

In their second study, Caglayan et al. used a home cage connected with a gating mechanism to the operant chamber to perform the stop-signal-task for assessing symptoms of attention deficit disorders and schizophrenia. The automated HCM approach required only minimal experimenter involvement, reduced training time for the mice by about 20%, and importantly, considerably improved task sensitivity.

### Fear and Anxiety

Fear and anxiety are commonly studied with short experiments that cannot be applied repeatedly, and therefore, do not allow the analysis of disease progression or the assessment of long-term treatment. Schuessler et al. customized an experimental chamber consisting of a safe nest and a foraging area with an operant lever, food port, water spout and shock grid floor. Foot shock was delivered specifically in the area where food and water were available. Using this “Risky Closed Economy” approach, fear and anxiety-related behavior were studied nearly 24/7 for extended periods as described, indicating flexibility based on decision-making when and how much to forage. This is a more naturalistic foraging scenario under controlled threat condition. Similarly, RFID-chipped transgenic mice in a partially automated HCM can be studied for their social, cognitive behavior in a complex environment that helps researchers identify the gene x environment interactions for affective and cognitive consequences of psychiatric disease as shown by Volkmann et al..

### Stroke

Most stroke models in rodents are induced *via* transient occlusion of the middle cerebral artery and are behaviorally characterized through standard motor tests. However, repetition of such tests might lead to habituation and changes in performance. Therefore, researchers tried to identify hallmarks after stroke induction on locomotor activity in HCM. A new open-source tracking software allowed calculating distance traveled, speed and also turning behavior since stroke models have the tendency to move more unilaterally if untreated. Here, recovery from stroke was not augmented by dietary induction as shown previously in humans by Shenk et al. A follow-up paper from the same group determined whether voluntary wheel running in the HCM would induce a faster recovery from stroke. In fact, stroke-induced mice with running wheel had a better recovery from stroke in terms of motor skills as well as increased functional connectivity, cerebral blood flow, and vascular quality as reported by Lohkamp et al..

## Viral Infection Symptoms

**Figure 1:**
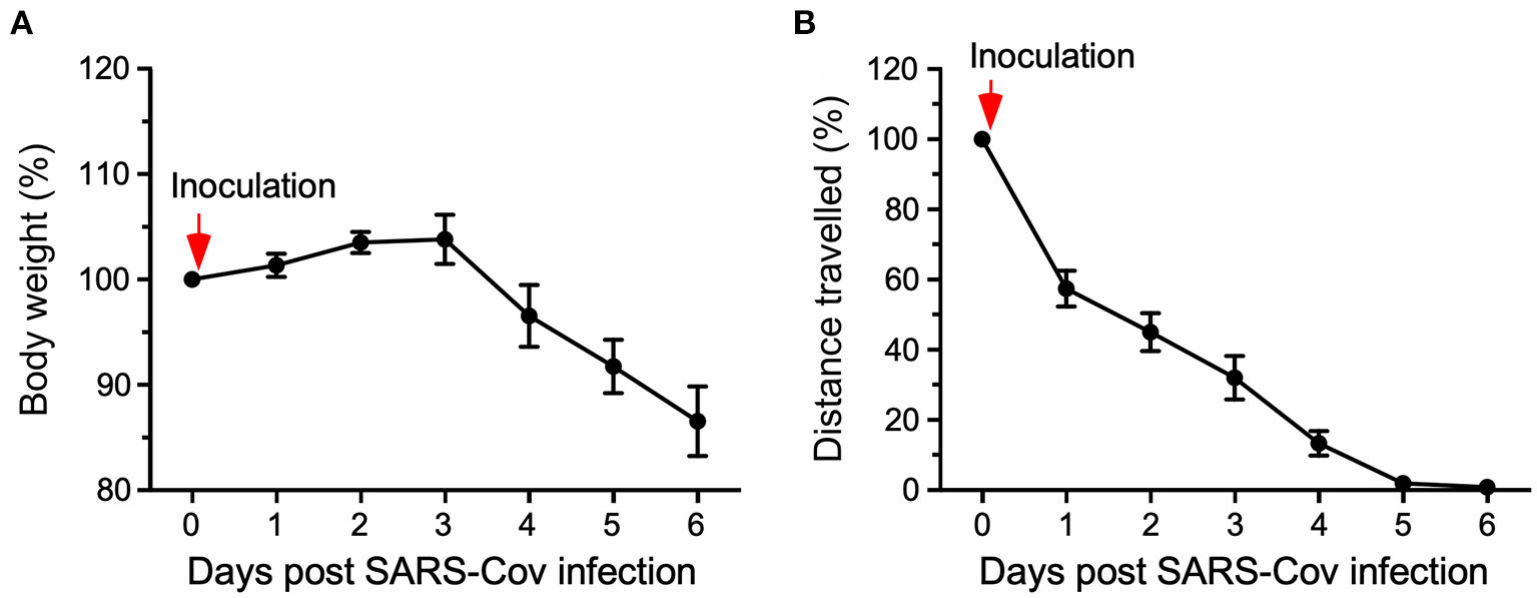
Eight single-housed K18-hACE2 (C57Bl/6J background) mice were exposed to the SARS-CoV-2 virus (2.8 × 10^4^ TCID50/ml). Changes in body weight **(A)** and total distance traveled **(B)** were assessed for six days relative to pre-inoculation values (100%). After viral exposure (inoculation), body weight dropped significantly on day 4, whereas the distance traveled was already significantly reduced by 30% on day 1 and reduced by almost 90% on day 4 indicating substantially higher sensitivity of locomotor activity than body weight change (Gaburro, 2021; modified from Kaufmann et al., 2022).

HCM also served in the evaluation of mice exposed to SARS-CoV-2. K18-hACE2 transgenic mice, harboring the humanized angiotensin-converting enzyme 2 (ACE2) receptor to which the spike protein binds, were used to assess the pathophysiology induced by SARS-Cov-2. Typically, in infectious disease studies using mice both viral titer and body weight are monitored. However, SARS-Cov-2 infections in humans cause fatigue rather than body weight loss. Using a HCM system, K18-hACE2 transgenic mice infected with SARS-CoV-2 were evaluated daily for body weight as well as locomotor activity (see Figure 1). These results demonstrate the potential of how automated HCM can aid both, animal welfare assessment and viral infection-induced symptom detection of disease in mice similar as in humans (Gaburro, 2021; Kaufmann et al., 2022).

## Energy Expenditure and Circadian Locomotor Activity

A survey of 30 mouse strains by König et al. indicated significant effects of strain, gender and circadian phase on voluntary physical activity and energy expenditure in automated HCM. This study highlights that naturally occurring genetic variation modulates various innate activity behaviors, food intake and energy expenditure in mouse strains.

## From HCM Based-Exploration of Mice Back to Human Diagnostic

An intriguing development originates from phenotyping studies of predominantly mouse behavior based on the long-standing collaboration between Ilan Golani and Yoav Benjamini (e.g., Fonio et al., 2009) consisting of computational exploratory data analysis methodology including videotaping, tracking, and customized data analysis. This has now been applied to human pre-walking infants in the interdisciplinary approach by Frostig et al. aimed to examine the organization of infant exploration in a novel setting. Here, the stationary mother serves as “home-base” reference point to characterize the exploration patterns of typically developing infants, but does not serve, or serves much less as a reference, for non-typically developing infants. This approach identified profound differences between typically and non-typically developing human infants with respect to their excursions from the mother as important reference point. Thus, this approach may serve as novel assay to screen for deviating child development with very early diagnostic potential at 8–18 months, as observed in autism spectrum disorder, if replicated and extended on a larger scale.

## Conclusion

The interest toward the Research Topic on HCM and the still widely diverging avenues of development suggest that this field is just at the beginning of expansion and far from maturation. This general approach could set the basis for novel studies in which digital libraries can be constructed for control animals which eventually could contribute to a reduction of animal use in research. HCM approaches will provide for a better characterization of normal vs. deviant behavior including better “symptom progression” and earlier recognition of potential diagnostic features for improved animal welfare and science.

## Author Contributions

SG, OS, YW, and JK wrote the manuscript and revised it. ML revised the manuscript. All authors contributed to the article and approved the submitted version.

## Funding

This work was supported by a NIH grant MH099073 (JK).

## Conflict of Interest

SG works as scientific director at Tecniplast S.p.A. ML works as Chief Executive Officer at Sylics BV. The remaining authors declare that the research was conducted in the absence of any commercial or financial relationships that could be construed as a potential conflict of interest.

## Publisher's Note

All claims expressed in this article are solely those of the authors and do not necessarily represent those of their affiliated organizations, or those of the publisher, the editors and the reviewers. Any product that may be evaluated in this article, or claim that may be made by its manufacturer, is not guaranteed or endorsed by the publisher.
